# Broad-Spectrum Inhibitors against Class A, B, and C Type β-Lactamases to Block the Hydrolysis against Antibiotics: Kinetics and Structural Characterization

**DOI:** 10.1128/spectrum.00450-22

**Published:** 2022-09-07

**Authors:** Nabeela Farhat, Divya Gupta, Abid Ali, Yogesh Kumar, Farheen Akhtar, Senthilguru Kulanthaivel, Prashant Mishra, Feroz Khan, Asad U. Khan

**Affiliations:** a Medical Microbiology and Molecular Biology Lab, Interdisciplinary Biotechnology Unit, Aligarh Muslim University, Aligarh, Uttar Pradesh, India; b Department of Life Sciences, Uttarakhand Technical University, Dehradun, Uttarakhand, India; c CSIR-Central Institute of Medicinal & Aromatic Plants, CIMAP, Lucknow, Uttar Pradesh, India; d Department of Biochemical Engineering and Biotechnology, Indian Institute of Technology, New Delhi, Delhi, India; University of Texas Southwestern Medical Center

**Keywords:** antibiotic resistance, MDR, β-lactam antibiotic, β-lactamases, β-lactamase inhibitors, NDM-1, antimicrobial agents, inhibitors

## Abstract

The emergence of antibiotic resistance has led to a global crisis for the physician to handle infection control issues. All antibiotics, including colistin, have lost efficiency against emerging drug-resistant bacterial strains due to the production of metallo-β-lactamases (MBLs) and serine-β-lactamases (SBLs). Therefore, it is of the utmost importance to design inhibitors against these enzymes to block the hydrolytic action against antibiotics being used. Although various novel β-lactamase inhibitors are being authorized or are under clinical studies, the coverage of their activity spectrum does not include MDR organisms expressing multiple classes of β-lactamases at a single time. This study reports three novel broad-spectrum inhibitors effective against both SBLs and MBLs. Virtual screening, molecular docking, molecular dynamics simulations, and an *in silico* pharmacokinetic study were performed to identify the lead molecules with broad-spectrum ability to inhibit the hydrolysis of β-lactam. The selected compounds were further assessed by *in vitro* cell assays (MIC, 50% inhibitory concentration [IC_50_], kinetics, and fluorescence against class A, B, and C type β-lactamases) to confirm their efficacies. A 3-(4,5-dimethylthiazol-2-yl)-2,5-diphenyl tetrazolium bromide (MTT) assay was performed to check the toxicity of screened lead molecules. All three selected inhibitors were found to reduce MIC and showed good affinity against all the SBLs and MBLs produced by class A, B, and C type β-lactamases. These nontoxic novel non-β-lactam broad-spectrum inhibitors bind to the active site residues of selected β-lactamases, which are crucial for β-lactam antibiotic hydrolysis. These inhibitors may be proposed as a future drug candidate in combination with antibiotics as a single formulation to control infection caused by resistant strains. Hence, this study plays a significant role in the cure of infections caused by antibiotic-resistant bacteria.

**IMPORTANCE** Several inhibitors for usage in conjunction with antibiotics have been developed. However, to date, there is no commercially available broad-spectrum β-lactamase inhibitor that targets both MBLs and SBLs. Here, we showed three novel broad-spectrum inhibitors with promising results through computational techniques and *in vitro* studies. These inhibitors are effective against both SBLs and MBLs and hence could be used as future drug candidates to treat infections caused by multidrug-resistant bacteria producing both types of enzymes (SBLs and MBLs).

## INTRODUCTION

β-lactamases have the ability to degrade almost all classes of β-lactam drugs (1). Ambler-classified β-lactamases are categorized in four classes on the basis of structural or molecular relationships: class A, class B, class C, and class D. Class A, C, and D β-lactamases are serine β-lactamases, as they utilize serine residue on their active site to perform hydrolysis of β-lactam drugs. Class B β-lactamases are recognized as metalloenzymes, as they involve divalent zinc ions to carry out the hydrolysis of drugs (2). Gram-negative microorganisms with multidrug resistance (MDR) and ever-increasing substrate specificity dispersing rapidly across the world represent an alarming stage of the fight against antibiotic resistance. This expanding predominance of multidrug resistant (MDR), extensively drug-resistant (XDR), and pan drug-resistant (PDR) microbes has worsened the situation to a greater extent, ultimately augmenting human illness, suffering, and death, as well as increasing the cost and length of treatments (3). The limited options of antibiotics compel researchers to identify novel methods to fight against antibiotic resistance. One of the novel approaches is the application of an inhibitor molecule in combination with an antibiotic, so that the inhibitor can block the enzymatic activity of β-lactamases while the antibiotic can carry out its bactericidal effect at the same time. In view of this, some inhibitors have been developed for use in conjunction with antibiotics. Though sulbactam, tazobactam, and clavulanate are powerful β-lactamase inhibitors, their viability is confined to class A β-lactamases. To date, there is no commercially available broad-spectrum β-lactamase inhibitor that covers metallo-β-lactamases (MBLs) and serine-β-lactamases (SBLs) simultaneously. However, some have reached clinical trials. One of the serious issues to be handled these days is the coexpression and production of MBLs and SBLs together in antibiotic-resistant microbes. Hence, there is an urgent need to discover broad-spectrum β-lactamase inhibitors. This additionally incorporates all subclasses of MBLs, where an inhibitor with good drug ability and approximately null activity against mammalian zinc-containing enzymes, such as alcohol dehydrogenase or carboxypeptidase A, needs to be discovered. There are a few β-lactamase inhibitors being worked on; however, just a couple of them can restrain class D and even fewer class B MBLs. Very few novel β-lactamase inhibitors, including avibactam and sulbactam, are clinically accessible, and others are undergoing different stages of clinical trials. Vaborbactam (earlier known as RPX7009) and tazobactam are FDA-approved inhibitors. The phase II clinical trial for the structural analog of tazobactam, enmetazobactam (formerly known as AAI101) was successfully completed (EudraCT number in EU clinical trials register: 2016-005161-31). Penicillin sulfone inhibitor LN-1-255 also inhibits multiple classes of SBLs, such as SHV-1 and SHV-2 (class A). Relebactam is effective against MBLs of class B and OXA-type enzymes of class D. Other β-lactamase inhibitors are reported, such as nacubactam (formerly known as OP0595, an aminoethoxy-substituted analogue of avibactam), ETX2514 (a reversible DBO inhibitor), VNRX-5133 (taniborbactam, a new boronic acid BLI), and ANT431 (a novel pyridine-2-carboxylic acid).

The strategy for increasing the efficacy of the antibiotics against β-lactamases is designing inhibitors (4). The proposed hypothesis in this study is to design non-β-lactam broad-spectrum inhibitors which will reduce the chances of resistance against these inhibitors (5). Hence, in this study, we have screened the Maybridge database against all the classes of β-lactamase and screened compounds to be validated by a computational method as well as *in vitro* cell assays (MIC, 50% inhibitory concentration [IC_50_], kinetics, and fluorescence) for the development of broad-spectrum inhibitors.

## RESULTS

### *In silico* docking.

**FIG 1:**
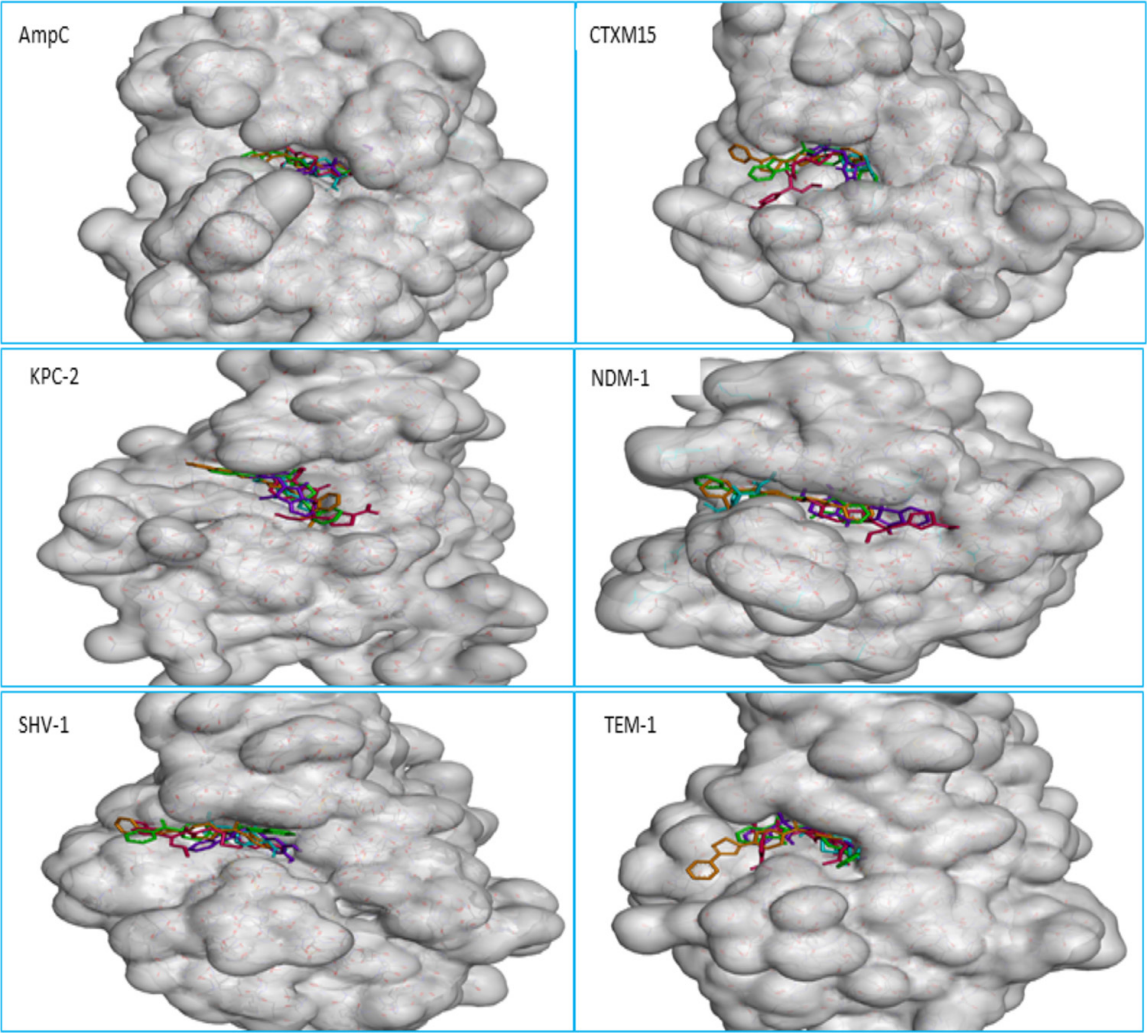
All selected ligands, avibactam, and cefotaxime bind to the same active site of the enzyme. Sky blue, avibactam; red, cefotaxime; purple, D63green D2148; orange, D2573.

Virtual screening is one of the best approaches to reduce the time and cost restrictions of drug development. Hence, we have employed this methodology to recognize the promising lead molecules which are probably active against preferred enzyme targets. GOLD (Genetic Optimization for Ligand Docking) software was utilized to perform virtual screening of ligands retrieved from the Maybridge database. The GOLD fitness score of docked complexes along with molecular interactions between ligand and the specific target were used for screening promising drug candidates. Five compounds were shown to have a good binding score with all selected enzyme targets compared with others: M63 (2-[2-(3,4-dihydroxy phenyl)-2-oxoethyl]-1H-1,2-benzisothiazole-1,1,3[2H]-trione), M1729 (2-[(2-hydroxyethyl) amino]-6-[phenylthio]-2,3-dihydro-1H-benzo[de]isoquinoline-1,3-dione), M2148 (2-[2-(4-benzoylpiperidino)-2-oxoethyl]-1H-isoindole-1,3[2H]-dione), M2573 (2-{[4-(3-phenyl-1,2,4-thiadiazol-5-yl)piperazino]carbonyl}benzoic acid), and M2627 [2-(2-oxo-2-{4-[2-(trifluoromethyl)-4-quinolinyl]piperazino}ethoxy)acetic acid]. Also, the GOLD score of these five compounds with all selected enzymes was higher than that of the commercially available inhibitor (avibactam) and antibiotic (cefotaxime) as shown in Table 1. To diminish false selection, the selected compounds along with standard inhibitors and antibiotics were docked against representative β-lactamases of classes A, B, and C using AutoDock Vina software. This led to the identification of three potential lead drug candidates with a high GOLD score, high binding energy, and firm binding ability. The study of hydrogen bond profile, hydrophobic interactions, and other bonds such as a pi-sulfur bond, the electrostatic bond between selected ligands and representative β-lactamases of classes A, B, and C was done precisely using Discovery Studio software (Table 2). The docked complexes were assessed precisely for favorable binding, high binding energy, and firm interaction with the active site residues of the target enzymes using Discovery Studio software (Fig. 1). All the compounds along with the inhibitor and antibiotic were found to bind to the same active site of the selected β-lactamase enzymes (Fig. 1). These three compounds were selected for further analysis.

**TABLE 1:**
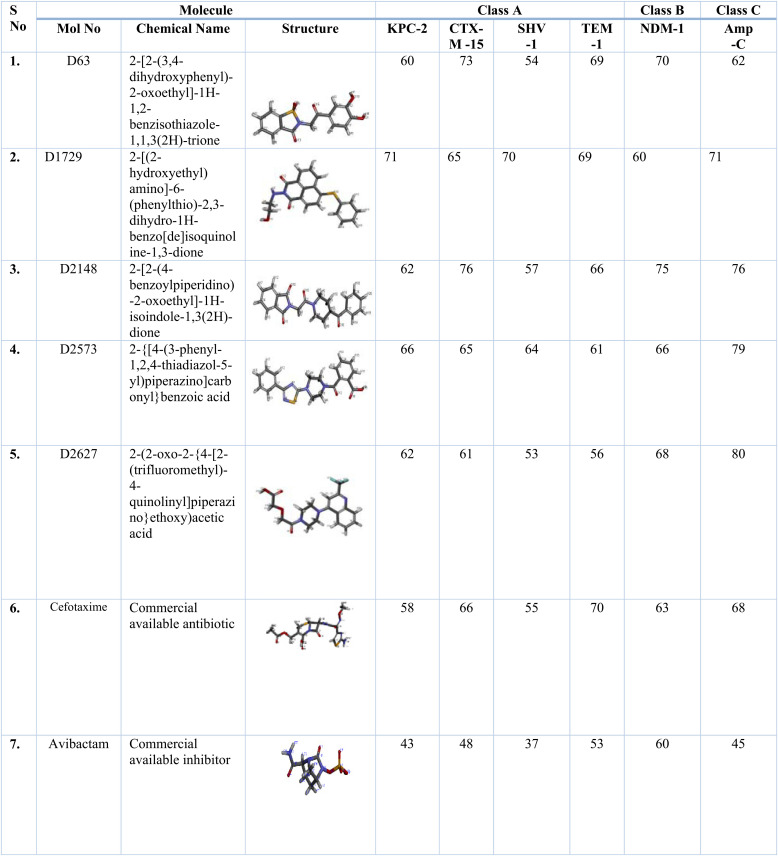
GOLD fitness score of five selected lead compounds, cefotaxime, and avibactam

**TABLE 2:** Detail of binding energy interpreted using AutoDock Vina software along with brief description of key active site residues involved in binding

β-Lactamase variant	Attribute	Data for:				
		D2573	D2148	D63	Cefotaxime	Avibactam
KPC-2	Binding energy	–9.1	–8.6	–8.1	–7.3	–7.2
	H bond	Ser70, Ser130, Ser170, Arg220, Thr235, Thr237	Ser70, Asn132, Asn170, Thr235	Ser70, Lys73, Ser130, Asn132, Glu166, Asn170, Thr237	Ser70, Ser130, Thr215, Arg220, Thr235, Thr237	Ser70, Trp105, Ser130, Asn132, Glu166, Thr237
	Hydrophobic bond	Trp105, Leu167, Thr216	Trp105, Leu167	Trp105		Trp105
	Other bond				His219 (Pi sulfur bond)	
CTXM -15	Binding energy	–9.2	–9.1	–8.6	–8.4	–6.9
	H bond	Ser70, Asn104, Ser130, Thr235, Ser237	Asn104, Asn132, Asn170, Thr235, Ser237	Ser70, Ser130, Asn132, Thr235, Gly236	Ser70, Asn132, Lys234, Thr235, Ser237, Pro268, Ala270	Ser70, Ser130, Asn132, Lys234, Ser237, Thr235
	Hydrophobic bond	Tyr 105, Pro167	Tyr105, Gly238-Gly239	Arg274		
SHV-1	Binding energy	–8.0	–7.5	–7.2	–7.2	–6.8
	H bond	Ser70, Asn132, Ala237	Ser70, Ser130, Thr167, Ala237	Ser70, Ser130, Asn132, Asn170, Thr235	Ser70, Ser130, Asn132, Thr167, Asn170, Lys234	Ser70, Ser130, Asn132, Asn170, Thr235
	Hydrophobic bond	Glu168, Ala237	Tyr105	Val216, Ala237	Ala237	Ala237
	Other bond	Glu168 (electrostatic bond), Glu240 (Electrostatic bond)	Glu240 (electrostatic bond)		Glu240 (electrostatic bond)	
TEM-1	Binding energy	–8.2	–8.2	–8.3	–8.0	–7.3
	H bond	Ser70, Lys73, Ser130, Asn132	Ser70, Asn132, Asn170, Ser235, Arg244	Ser70, Ser130, Asn132, Asn170, Val216, Ser235, Arg244	Ser70, Ser130, Pro167, Asn170, Ser235, Ala237, Gly238, Arg244	Lys 73, Ser130, Asn132, Arg244
	Hydrophobic bond	Tyr105, Ala237	Val216, Ala237, Ala237-Gly238, Gly238-Glu240	Val216, Ala237, Ala237-Gly238,	Tyr105, Ala237	Tyr105, Val216, Ala237
	Other bond			Glu104 (electrostatic bond), Glu166 (electrostatic bond)	Tyr105 (Pi-sulfur bond)	
NDM-1	Binding energy	–8.8	–9.5	–8.1	–6.7	–6.3
	H bond	Gly36, His250	Asn220, Ser251, His189	His122, Gln123, Asp124, Lys211, Asn220	Gln123, Glu152, His189, Lys211, Asn220, His250	Lys211, Gly219
	Hydrophobic bond	ILE35, Ala215, His250	Ile35, Lys211, Ala215, Lys216, His250	His122, His250	Ile35, His122, His250	ILE35, Val73, His250
	Other bond	Zn1: Zn2 (electrostatic bond), Asp124 (electrostatic bond), Asp212 (electrosatic bond)	Zn2 (electrostatic bond)	Zn2 (electrostatic bond), His122 (electrostatic bond), Asp124 (electrostatic bond)	Zn1 (metal acceptor bond), Trp93 (Pi-sulfur bond)	Zn1: Zn2 (metal acceptor bond), His189
Amp-C	Binding energy	–9.7	–9.9	–8.5	–8.9	–6.9
	H bond	Gln 146, Asn 179, Thr343, Asn373	Tyr177, Asn179, Thr 343, Asn 373	Ser90, Arg175, Tyr177, Gly313, Thr343, Asn373	Ser 90, Asn 179, Gly313, Thr316, Lys342, Thr343, Ser345, Asn373, Glu 299, Asn 314	Ser 90, Asn 179, Ser345, Asn 373
	Hydrophobic bond	Ala 319, Met 318, Tyr177, Tyr 249, Ala319	Ala319, Tyr249	Tyr 177, Ala 319	Tyr 177, Thr249, Ala 319	Tyr 177, Ala 319
	Other bond	Glu 299 (electrostatic bond)		Glu 299 (electrostatic bond)	Tyr 177	

### Binding modes refined by molecular dynamics simulation.

**FIG 2:**
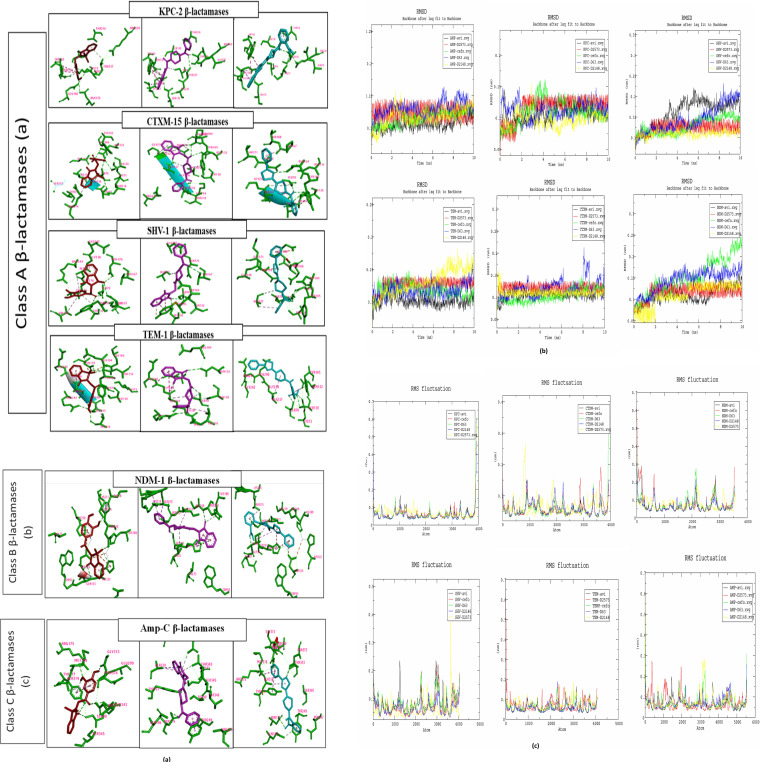
(a) Description of binding site molecular interactions between enzymes and ligands along with key amino acid residues of targeted enzymes involved in binding with D63, D2148, and D2573. Red, D63; purple, D2148; sky blue, D2573. (b) Root mean square deviation (RMSD) was done for backbone atoms of docked complexes at 300K. Black, avibactam; red, D2573; green, cefotaxime; blue, D63; yellow, D2148. (c) Root mean square fluctuation (RMSF) was done for backbone atoms of docked complexes at 300K. Black, avibactam; red, D2573; green, cefotaxime; blue, D63; yellow, D2148.

The molecular dynamics (MD) studies were performed using the Groningen Machine for Chemical Simulation (GROMACS) up to 10 ns (for each ligand-bound β-lactamase system) and 50 ns (for the avibactam and D2573-bound β-lactamase system; because it showed the best AutoDock binding energy with all the proteins). All the complexes were prepared and run for MD simulations. The simulation of 10 ns was carried out separately for all the protein-ligand complexes. The root mean square deviation (RMSD) was calculated for backbone atoms’ positional differences over time for different classes of β-lactamase with each compound as shown in Fig. 2. The stable RMSD was obtained for the protein complexes of CTX-M-15 followed by Amp C, KPC-2, NDM-1, SHV-1, and TEM-1. The stability of the protein-ligand complex was calculated to check the effective confirmation sampling and conformational stability. The RMSD calculation was performed to find the binding stability using Gromacs software. The stability of the protein related to its conformational change and deviation produced throughout the course of the simulation was determined, which reveals that the smaller the deviation, the more stable the protein is. The results reveal that compound D2573 shows less RMSD deviation than the other compounds when run for 10 ns for all selected β-lactamases (Fig. 2). The root mean square fluctuations (RMSF) of 10 ns were generated to understand the movement of compounds’ flexible regions; it is a measure of the displacement of a particular atom, or group of atoms, relative to the reference structure, averaged over the number of atoms (Fig. 2).

**FIG 3:**
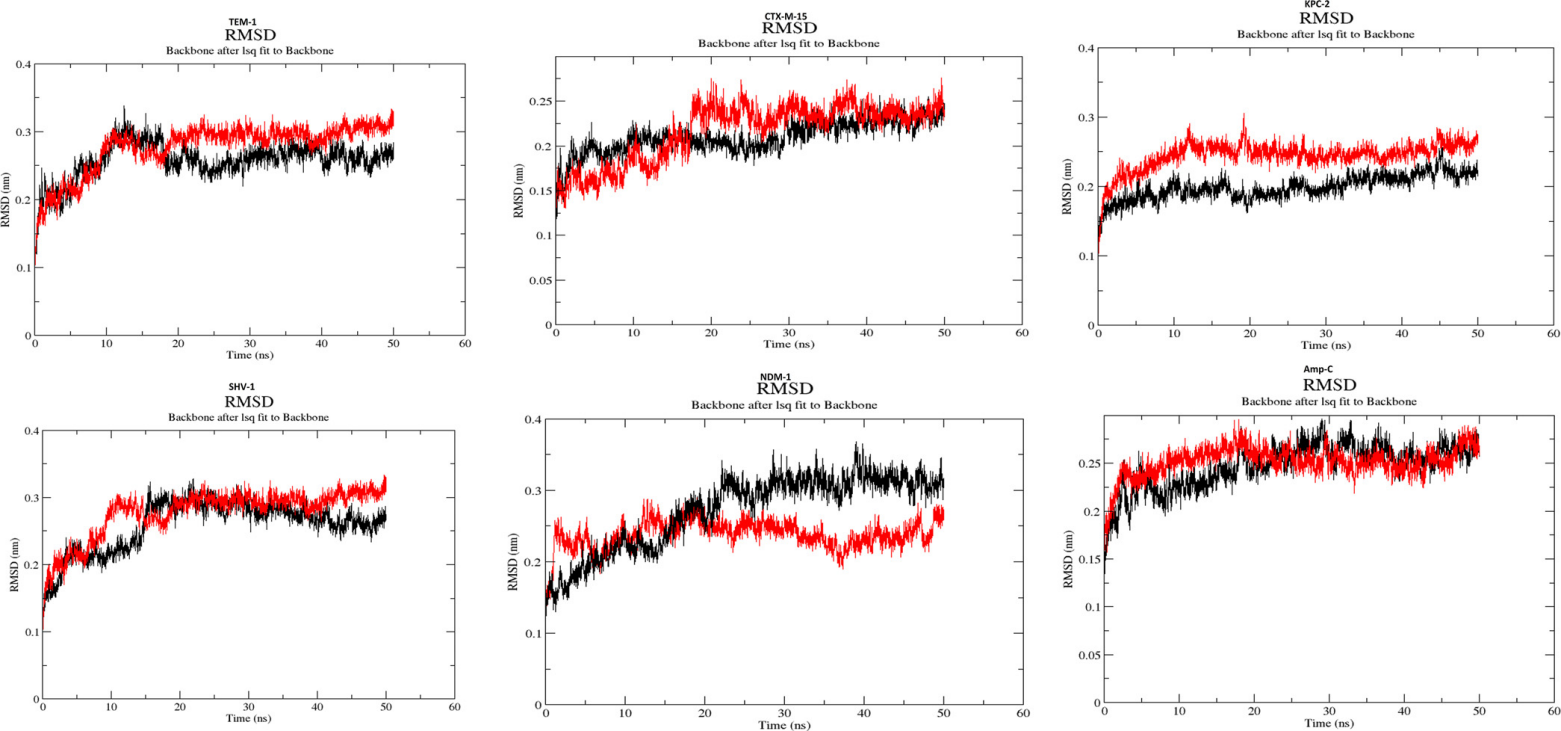
Backbone root mean square deviation (RMSD) values in nanometers (*y* axis) along with the time frame in nanoseconds (*x* axis) Black, avibactam; red, D2573.

All the docked complexes’ trajectories remained stable with proper conformations during the 50-ns simulation, showing the stability of ligands during interaction with the protein active site (Fig. 3). With NDM-1, the RMSD of D2573 is lower than that of avibactam. The minimal RMSD value of backbone atoms strongly predicts that the docked complex will have stable dynamic behavior.

### *In silico* pharmacokinetic study.

The drug-likeness properties of the final lead compounds were compared with commercially available inhibitors after getting promising results from docking and simulation studies. This was done to ensure the compliance of the pharmacokinetic properties of the selected ligands with the standard range. Table 3 shows that the selected ligands follow Lipinski’s rule of five and must have good solubility, permeability, and oral bioavailability.

**TABLE 3:** Comparison of physicochemical properties of selected ligands and commercial inhibitor with computational parameters of drug likeness (oral bioavailability) through Lipinski’s rule of five

Selected ligand	No. of H bond donors (≤5)	No. of H bond acceptors (≤10)	Mol wt (≤500)	CLogP (≤5)	No. of rotatable bonds (≤5)
D63	2	7	333.32	1.45	3
D2148	0	6	376.41	2.17	4
D2573	1	7	394.45	2.38	4
Avibactam	2	6	265.25	−1.8	3

Pharmacokinetic prediction of the selected ligands was done using the SwissADME online serve tool (http://swissadme.ch/) and PreADMET (http://preadmet.bmdrc.org/) (Table 4). The data revealed that selected ligands strongly bind with the plasma proteins compared to avibactam, which binds weakly with the plasma proteins. Toxicity prediction of all ligands of interest was done using ADMET modules in Discovery Studio 3.5 software (Accelrys, USA) and the PreADMET online server (Table 4).

**TABLE 4:** Pharmacokinetic and toxicity prediction for selected ligands

Parameter	Data for:			
	D63	D2148	D2573	Avibactam
BBB permeant	No	No	No	No
Plasma protein binding prediction	91.97	89.047	90.77	46.25
Caco2cell permeability	1.59	21.92	20.87	15.35
GI absorption	High	High	High	Low
P-glycoprotein inhibition	None	None	None	None
CYP3A4 inhibition	No	Yes	Yes	No
CYP2C19 inhibition	No	Yes	Yes	No
CYP2C9 inhibition	No	Yes	Yes	No
CYP2D6 inhibition	No	No	No	No
Ames test	Nonmutagen	Nonmutagen	Nonmutagen	Nonmutagen
Skin irritancy	None	None	None	None
Ocular irritancy	Mild	Mild	Mild	Moderate
Aerobic biodegradability	Nondegradable	Degradable	Nondegradable	Degradable
Hepatotoxicity	True	True	True	True
Carcino_mouse	Negative	Negative	Negative	Negative
Carcino_rat	Negative	Negative	Negative	Negative

### MIC and MBC determination.

The *bla*_SHV-1_, *bla*_TEM-1_, *bla*_KPC-2_, and *bla*_Amp-C_ genes were cloned in the pET-28a vector individually, followed by its transformation in Escherichia coli *DH5α* cells. The MIC values was determined for clinical strains (AK-10, AK-12, AK-18, NP-6, AK-67, and AK-66) and E. coli *DH5α* cells with selected clones (*bla*_SHV-1_, *bla*_TEM-1_, *bla*_KPC-2_, *bla*_Amp-C_, *bla*_CTX-M-15_, and *bla*_NDM-1_) carrying vectors individually. The MIC values are listed in Table 5. All of the clones had increased MICs for the antibiotics employed in this study. However, when these antibiotics (excluding cefoxitin) were combined with novel screened compounds, their MICs were reduced. The reduction in MIC for the NDM-1-producing clone was observed with imipenem and meropenem when combined with each of these molecules, i.e., D63, D2148, and D2573. The reduction in MICs of imipenem and meropenem in combination with these inhibitors against the clinically significant NDM-1-producing clone are of paramount importance, as most of the broad-spectrum inhibitors are not effective against MBLs. The minimum bactericidal concentration (MBC) values of antibiotic-inhibitor combinations against clinical strains and clones with each marker are listed in Table 6.

**TABLE 5:** MIC values of antibiotics and antibiotic-inhibitor combinations for E. coli DH5α transformed with recombinant β-lactamase enzyme

Antibiotic or antibiotic + inhibitor	MIC (μg/mL) for recombinant clones/clinical strains of:											
	CTX-M-15	AK-10	tEM-1	AK-12	SHV-1	AK-18	KPC-2	NP-6	Amp-C	AK-67	NDM-1	AK-66
Imipenem	32	128	16	64	16	64	16	64	16	1,024	512	1,024
Imipenem + D63	16	32	16	32	16	32	8	16	16	256	256	256
Imipenem + D2148	4	8	16	32	8	16	4	8	8	256	256	256
Imipenem + D2573	4	8	8	16	4	8	4	8	16	256	256	256
Meropenem	2	32	2	16	2	32	2	16	4	1,024	1,024	1,024
Meropenem + D63	1	8	1	4	2	16	1	4	1	256	256	256
Meropenem + D2148	1	8	1	8	1	4	1	4	1	256	256	256
Meropenem + D2573	1	8	1	4	1	4	1	4	2	512	512	512
Cefotaxime	512	1,024	2	16	4	32	2	16	2	512	256	512
Cefotaxime + D63	512	512	1	4	2	4	1	4	4	256	256	256
Cefotaxime + D2148	128	128	8	64	16	32	4	16	8	256	256	256
Cefotaxime + D2573	256	256	4	16	1	2	1	4	16	512	512	512
Cefoxitin	32	64	32	64	32	64	32	64	32	64	32	64
Cefoxitin + D63	64	64	64	128	8	8	8	8	64	128	128	128
Cefoxitin + D2148	64	128	64	128	64	64	64	64	64	128	128	128
Cefoxitin + D2573	64	128	32	64	8	8	32	32	64	128	128	128

**TABLE 6:** Minimum bactericidal concentrations (MBC) of antibiotic-inhibitor combinations after 24 h

Antibiotic or antibiotic + inhibitor	MBC (μg/mL) of recombinant clones of:					
	CTX-M-15	tEM-1	SHV-1	KPC-2	Amp-C	NDM-1
Imipenem + D63	16	16	16	8	16	256
Imipenem + D2148	8	16	16	4	8	256
Imipenem + D2573	4	8	16	4	16	256
Meropenem + D63	1	1	2	1	1	256
Meropenem + D2148	1	1	2	1	1	256
Meropenem + D2573	4	2	4	2	2	512
Cefotaxime + D63	512	2	2	1	4	256
Cefotaxime + D2148	512	8	16	8	8	256
Cefotaxime + D2573	256	8	2	1	16	512
Cefoxitin + D63	64	64	16	16	64	256
Cefoxitin + D2148	64	64	64	64	64	256
Cefoxitin + D2573	64	32	16	32	64	256

### IC_50_ value determination.

**FIG 4:**
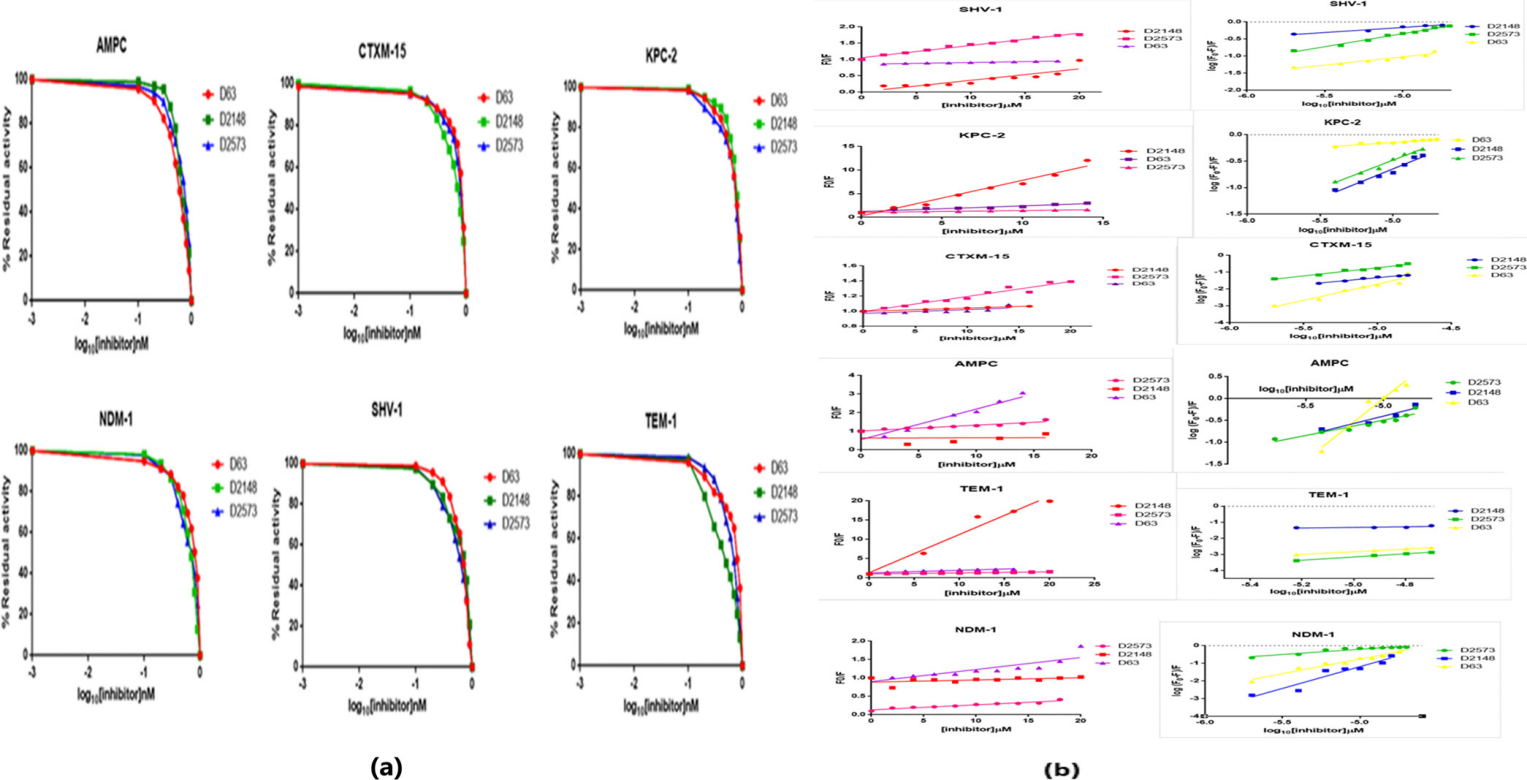
(a) Representation of IC_50_ values for all the three inhibitors with residual activity of all classes of protein, monitored by the hydrolysis of different concentrations of inhibitor with a constant value of 100 μM nitrocefin. (b) Stern-Volmer and modified stern Volmer plots. D2573, D2148, and D63 induced fluorescence quenching of NDM-1, SHV-1, AMPC, TEM-1, KPC-2, and CTXM-15 at 298 K.

The IC_50_ values of all the proteins were calculated to determine the efficacy of each inhibitor. The IC_50_ values were calculated for the proteins NDM-1, Amp-C, KPC-2, CTX-M-15, SHV-1, and TEM-1 with each of its inhibitors (D63, D2148, and D2573) to compare their efficacies at a fixed concentration 100 μΜ of nitrocefin (Table 7). The IC_50_ values for all protein-inhibitor combinations were found in the range of 0.449 to 0.779 nM as shown in Fig. 4a. The IC_50_ values for class A β-lactamase TEM-1 with D63 (0.726 nM), D2148 (0.449 nM), and D2573 (0.634 nM) are less than that for avibactam (8 nM). Similarly, CTX-M-15 with D63 (0.779 nM), D2148 (0.638 nM), and D2573 and (0.731 nM) and KPC-2 with D63 (0.695 nM), D2148 (0.744 nM), and D2573 (0.667 nM) have lower IC_50_ values than avibactam (CTX-M-15 [5nM] and KPC-2 [170 nM]). The SHV-1 IC_50_ values in combination with D63, D2148, and D2573 are 0.649 nM, 0.587 nM, and 0.655 nM, respectively. The IC_50_ values of the class C β-lactamase Amp-C with D63 (0.609 nM), D2148 (0.714 nM), and D2573 (0.655 nM) are lower than the to 128 nM value for avibactam (6). The class B β-lactamase NDM-1 IC_50_ values with D63, D2148, and D2573 are 0.733 nM, 0.632 nM, and 0.624 nM, respectively (Table 7).

**TABLE 7:** Concentration of inhibitor required to reduced enzyme activity by 50%

Inhibitor or protein	IC_50_ (nM) values for:		
	D63	D2148	D2573
TEM-1	0.726	0.449	0.634
SHV-1	0.649	0.587	0.655
CTX-M-15	0.779	0.638	0.731
KPC-2	0.695	0.744	0.667
NDM-1	0.733	0.632	0.624
Amp-C	0.609	0.714	0.655

### Steady-state kinetics parameter analysis.

**FIG 5:**
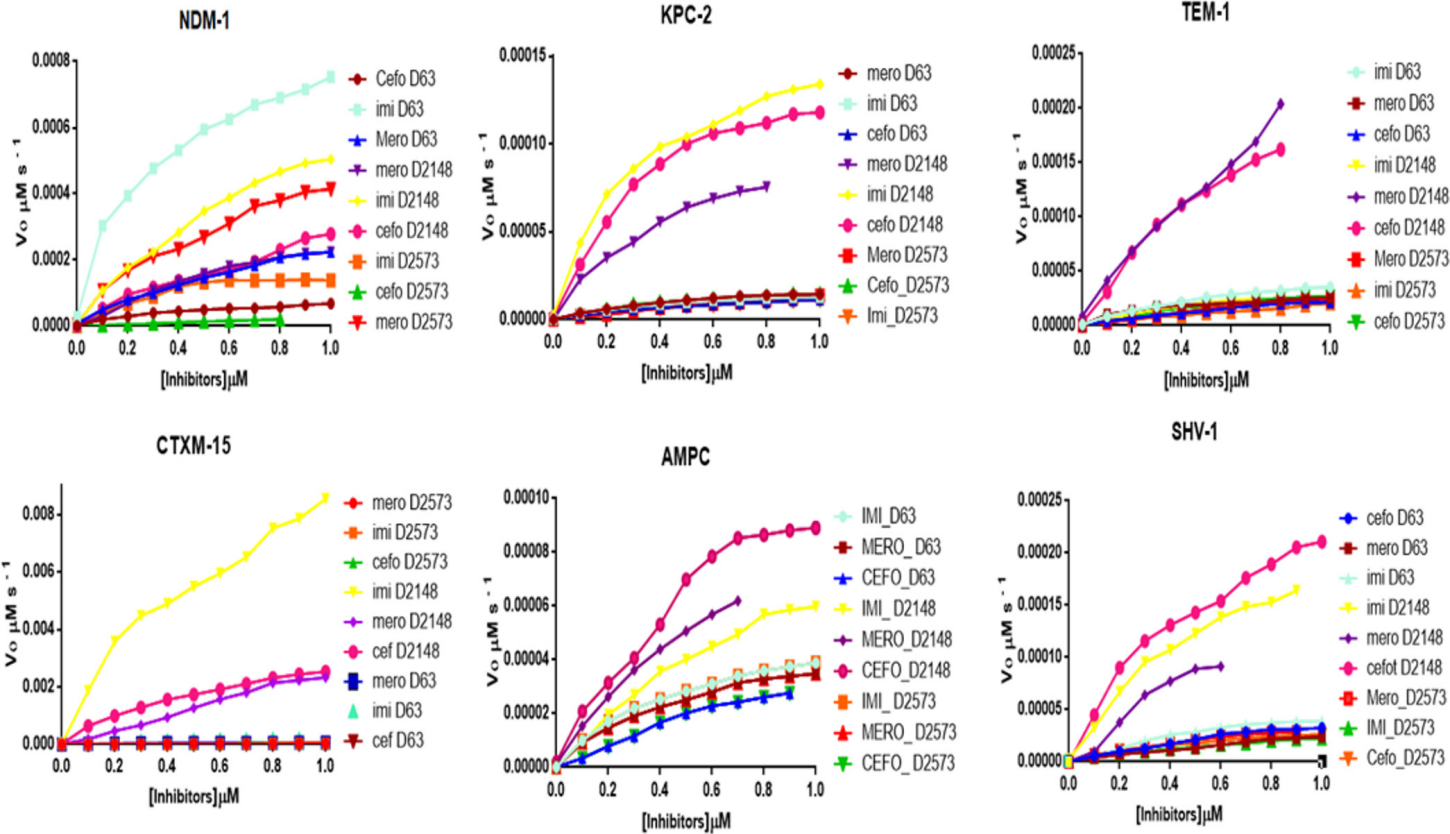
Determination of all three inhibitors and different antibiotics for NDM-1, SHV-1, Amp C, TEM-1, KPC-2, and CTX-M-15 represented the best fit (nonlinear regression).

The enzyme kinetics parameters (*K_m_*, and *k*_cat_) for all six proteins/enzymes in combination with different antibiotics (cefotaxime, imipenem, and meropenem), were calculated. The concentration of inhibitors, D63, D2148, and D2573, was as per the IC_50_ value (Fig. 5); the kinetic parameters are listed in Table 8. All the inhibitors showed similar affinity and catalytic activity as those shown by the known inhibitor, avibactam. However, in the case of NDM-1, a combination of imipenem plus inhibitors or meropenem plus inhibitors has shown better catalytic activity than that of avibactam.

**TABLE 8:** Enzyme kinetics parameter of NDM-1, SHV-1, AMP-C, TEM-1, KPC-2 and CTX-M-15 protein

Protein	Antibiotic or inhibitor	Data for imipenem:			Data for meropenem:			Data for cefotaxime:		
		*K*m or *K_i_* (μΜ)	*k*_cat_ (S^−1^)	*k*_cat_/*K_m_* (μM^−1^ s^−1^)	*K_m_* or *K_i_* (μΜ)	*k*_cat_ (S^−1^)	*k*_cat_/*K_m_* (μM^−1^ s^−1^)	*K_m_* or *K_i_* (μΜ)	*k*_cat_ (S^−1^)	*k*_cat_/*K_m_* (μM^−1^ s^−1^)
TEM-1	Avibactam	2,918	1.107	0.00037	12,315	5.916	0.00048	1,558	1.339	0.00085
	D63	673.1	1.194	0.00177	253.5	0.6169	0.0024	1,353	1.051	0.000776
	D2148	689.7	0.9853	0.0014	7,537	43.13	0.0057	757.7	6.332	0.0083
	D2573	2,665	1.454	0.00054	2,129	1.563	0.00073	905.2	1.056	0.0011
SHV-1	Avibactam	835.4	0.5531	0.000662	532	0.4803	0.000902	1,201	0.6119	0.000509
	D63	851	1.525	0.00179	2,881	1.928	0.000669	1,375	1.621	0.00117
	D2148	618.4	5.535	0.00895	1,376	6.436	0.00467	626.2	6.751	0.0107
	D2573	1,138	0.958	0.00084	979	1.232	0.00125	608	0.8431	0.0123
CTX-M-15	Avibactam	2,918	0.4807	0.0034	745.5	1.566	0.0021	2047	1.858	0.0009
	D63	480.5	5.572	0.011	228.4	1.723	0.0075	742.7	0.7320	0.00098
	D2148	675.9	2,720	4.0242	9,333	17,974	1.9258	670.6	838.6	1.2505
	D2573	2.212	1.454	0.00118	469.8	0.9091	0.00193	330.1	0.9055	0.0027
KPC-2	Avibactam	2,637	1.499	0.0005	1,740	0.72	0.0004	1299	0.549	0.00042
	D63	489.1	0.39	0.00079	511.7	0.45	0.00087	713	0.384	0.00053
	D2148	301.8	3.44	0.01139	491	2.47	0.00503	358.7	3.293	0.00918
	D2573	878.8	0.419	0.00047	2,786	1.02	0.00036	549.1	0.454	0.00082
NDM-1	Avibactam	615.5	2.194	0.0035	440.9	1.268	0.0028	212.2	1.036	0.0048
	D63	258.7	18.29	0.070	1,052	9.282	0.0088	410.8	1.833	0.0044
	D2148	995.4	20.6	0.020	986.4	9.201	0.0093	1,939	15.88	0.00818
	D2573	301.3	3.875	0.012	756.8	14.59	0.019	8,756	4.921	0.00055
Amp-C	Avibactam	321	0.861	0.002	1,442	2.213	0.0015	1,199	1.417	0.00118
	D63	499.22	1.158	0.0023	553.8	1.093	0.0019	1,522	1.537	0.0010
	D2148	1,136	2.631	0.002	794	2.637	0.003	796.6	3.401	0.0042
	D2573	499	1.158	0.002	553	1.09	0.0019	1,522	1.5	0.0009

### Fluorescence mechanism and binding affinity.

Fluorescence quenching can be either dynamic or static in nature. The fluorescence intensity was calculated to understand the mechanism of protein quenching from all three classes of β-lactamase with D63, D2148, and D2573. The values for binding constant (Kb) and K_SV_ are shown in Fig. 4b. The K_SV_ (Stern–Volmer quenching constant; [Q] is the concentration of inhibitor) of all protein-inhibitor complexes was calculated from the slope of plot Fo/F (fluorescence intensities in the absence and presence of quencher) versus [Q], whereas number of binding sites (*n*) and Kb values for all combinations of proteins and inhibitors were determined by the slope and intercept of plot log (Fo/F – 1) versus log[Q] (Table 9). In this study, the K_SV_ values were obtained, on the order of 10^2^ to 10^5^, for all enzyme-inhibitor complexes. Kb values were found on the order of 10^2^ of 10^6^ for all the combinations of proteins and inhibitors (Table 9).

**TABLE 9:** Binding parameters obtained from fluorescence quenching experiments

Protein	Inhibitor	K_SV_ (M^−1^)	Kb (M^−1^)	*n*	R^2^
TEM-1	D63	6.73 × 104	1.39 × 104	0.4298	0.8717
	D2148	1.02 × 106	3.32 × 104	0.2489	0.7735
	D2573	2.56 × 104	1.7 × 103	0.750	0.9722
SHV-1	D63	5.3 × 103	2.3 × 105	0.4791	0.9796
	D2148	3.5 × 104	2.06 × 105	0.2952	0.7756
	D2573	3.76 × 104	2.9 × 103	0.7621	0.985
CTX-M-15	D63	7 × 103	899 × 102	1.9313	0.6742
	D2148	4.3 × 103	2.6 × 102	1.163	0.992
	D2573	1.9 × 104	1.26 × 102	0.9674	0.9559
KPC-2	D63	8 × 102	0.13 × 105	0.33	0.7691
	D2148	6.59 × 105	33.2 × 102	1.148	0.9766
	D2573	3.84 × 104	15.6 × 102	1.1315	0.9913
NDM-1	D63	3.28 × 103	380 × 102	1.6673	0.7519
	D2148	9.7 × 103	1.25 × 106	2.457	0.552
	D2573	1.25 × 104	0.5 × 105	0.1842	0.9162
Amp-C	D63	1.64 × 105	1.52 × 102	0.8493	0.9153
	D2148	3.63 × 104	0.915 × 102	1.2792	0.9658
	D2573	3.13 × 104	1.07 × 103	0.7042	0.915

### Cytotoxicity.

**FIG 6:**
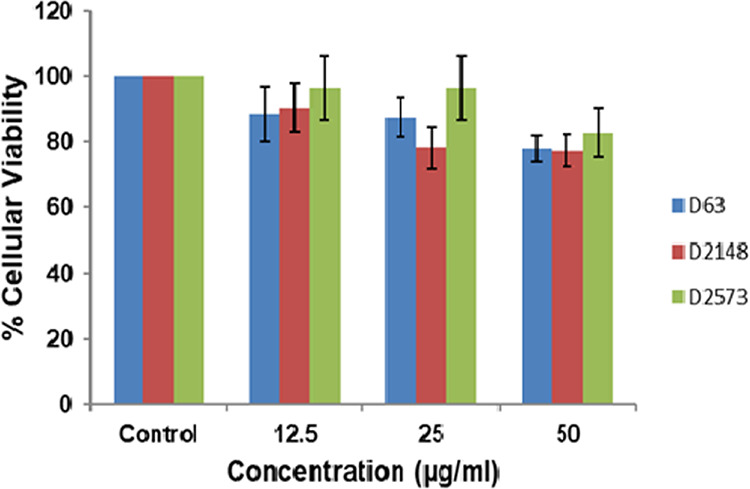
Effect of different concentrations of screened inhibitors on NIH 3T3 cells determined by MTT assay.

Cytotoxicity of lead molecules D2573, D2148, and D63 on NIH 3T3 cells was evaluated by MTT assay. The relative cellular viability of NIH 3T3 cells in the presence of each sample D2573, D2148, and D63 at different concentrations (12.5 μg/mL, 25 μg/mL, and 50 μg/mL) is shown in Fig. 6. Nearly 78% cellular viability was detected at the concentration of 50 μg/mL for samples D2148 and D63, whereas 82% of cells were found to be viable in sample D2573. Thus, the test concentration (25 μg/mL) was found to be nontoxic to NIH 3T3 cells.

## DISCUSSION

The latest effort to overcome the resistance of bacteria is a novel approach to design broad-spectrum non-β-lactam β-lactamase inhibitors, which are effective against many clinically relevant β-lactam groups of antibiotics.

The precise analysis of docked structure confirmed that some of the active site residues play a crucial role to establish a firm binding with the ligand molecule. In class A β-lactamases, Ser70, Ser 130, Asn 132, Thr 235, and Trp105/Tyr105 residues present in the active site help to form a strong interaction with antibiotics or inhibitors (7). Ser70, Ser 130, Asn 132, and Thr 235 induce hydrogen bonding because they fall under the category of polar uncharged amino acids. Trp105/Tyr105 mediate hydrophobic interactions because of their aromatic nature. (2, 8) In class B β-lactamases, their residues and zinc ions play important roles in binding. This is crucial to form hydrophobic interactions or hydrogen bonding, while metal ions assist to build an electrostatic bond (9, 10). In class C β-lactamases, Ser90, Asn179, Thr343, and Asn373 help to build hydrogen bonds, being polar uncharged amino acids. Tyr177 and Ala319 play a crucial role to form hydrophobic interactions. The enzymatic activity of β-lactamases can be inhibited by binding of inhibitor molecules to key residues of the enzymes, which ultimately helps to revitalize the bactericidal effects of traditional antibiotics if used in combination (Tables 1 and 2).

The MD simulation results reveal that compound D2573 shows less RMSD deviation than other compounds when run for 10 ns for all selected β-lactamases (Fig. 2). In the class A β-lactamases CTX-M-15, KPC-2, TEM-1, and SHV-1, D2573-bound complexes RMSD have less fluctuation. D2148- and D2573-bound NDM-1 complexes represent the most stable RMSD graph in comparison to others, while cefotaxime does not seem to be stably bound to NDM-1. In the case of Amp-C, D63 has moderately less stable binding, as evident from the RMSD plot.

In the MD simulation, the 50-ns run revealed that compound D2573 shows less RMSD deviation than avibactam for NDM-1 and the same deviation of avibactam in all the selected β-lactamases. The significant binding affinity and interaction between docked complexes were confirmed by RMSD analysis. It also revealed the stable behavior of docked complexes.

Pharmacokinetic features such as absorption, distribution, metabolism, and excretion (ADME) should be present in pharmacologically active compounds (11). A pharmacokinetic prediction study illustrates that selected ligands bind strongly with the plasma proteins compared to avibactam, which binds weakly with the plasma proteins ligands, and avibactam does not pass across the blood-brain barrier (BBB). D2148, D2573, and avibactam represent mild permeability for Caco2 cells, which helps to determine their intestinal absorption. D63 is the only compound showing low permeability for Caco2 cells. The intestinal absorption of the drug is very important for the bioavailability of the drug. The data revealed that gastrointestinal (GI) absorption is high for all selected ligands, whereas avibactam showed low GI absorption. Another important factor responsible for the absorption and disposition of drugs is plasma glycoprotein (P-gp) inhibition. Several studies have confirmed that the inhibition of P-gp is responsible for drug-drug interactions. All selected ligands and avibactam do not show any P-gp inhibition. CYP enzymes are found in the liver and are mainly responsible for drug metabolism and its elimination in the body. The most important CYP enzymes are CYP3A4, CYP2C19, CYP2C9, and CYP2D6. D2148 and D2573 showed little CYP3A4, CYP2C19, and CYP2C9 inhibition. Toxicity prediction is crucial to determining the mutagenicity and carcinogenicity of the compounds. None of the ligand molecules show any kind of toxicity or mutagenicity.

The MIC of meropenem was found to be reduced 2-fold when used in combination with D2573 in all selected β-lactamase enzymes, indicating the effect of inhibitors on antibiotic resistance. The combination of D2573 with imipenem also reduced MIC values in all β-lactamases except for Amp C, where no change in the MIC value was found in comparison with the control. In the case of D2148, the MIC of meropenem was found to be reduced 2-fold for class A β-lactamases and 4-fold for class B and C β-lactamases. D2148 also showed good inhibitory activity with imipenem, except for TEM-1, for which it does not affect MIC in any way. D63 also shows good inhibitory activity with meropenem. The MIC of class A β-lactamases was found to be reduced 2-fold, while the MIC of class B and C β-lactamases was found to be reduced 4-fold. D63 with imipenem represents decreasing MIC in CTX-M-15-, KPC-2-, and NDM-1-producing clones, while the same combination did not show any effect in MIC for TEM-1, SHV-1, and Amp-C. The combination of D2573 with cephalosporins (cefotaxime plus D2573 and cefoxitin plus D2573) or the combination of D2148 with cephalosporins (cefotaxime plus D2148 and cefoxitin plus D2148) or the combination of D63 with cephalosporins (cefotaxime plus D63 and cefoxitin plus D63) had higher MICs than the control. This shows that none of the compounds could be used in combination with cephalosporins. The MBC data also showed the same pattern as that of the MIC. The MIC and MBC data are in harmony with *in silico* data which concluded that three potential inhibitors, D2573, D2148, and D63, are potential candidates against broad-spectrum β-lactamase-producing bacterial strains.

The IC_50_ values of lead molecules, D2573, D2148, and D63, are lower than those of avibactam identifying them as ideal inhibitors for all classes of β-lactamase.

The enzyme kinetics parameter showed good affinity in class A β-lactamases (CTX-M-15, KPC-2, SHV-1, and TEM-1) and the class C β-lactamase Amp-C with all the antibiotics, imipenem, meropenem, and cefotaxime, i.e., similar to avibactam. However, for class B β-lactamase, these inhibitors (D2573, D2148, and D63) show good results compared to avibactam with imipenem and meropenem, while with cefotaxime, not much difference was observed. The catalytic efficiency of all the class A (CTX-M-15, KPC-2, SHV-1, TEM-1) and class C (Amp-C) β-lactamases are the same as that of avibactam with all the antibiotics (imipenem, meropenem, and cefotaxime), but in class B (NDM-1) all the inhibitors in combination with imipenem and meropenem show good catalytic efficiency compared to avibactam (Table 8).

The mechanism of enzyme-inhibitor interaction was determined by protein intrinsic fluorescence quenching measurement (12–14). Quenching mechanisms are of two types, dynamic (interacts indirectly with molecule) and static (forms ground-state complex) (15). The decrease or increase in hydrophobicity around Trp and Tyr residues is indicated by the blue and red shifts, respectively (16). Fluorescence results indicate that quenching is caused by the formation of a strong complex between all of the protein-inhibitor complexes, rather than by dynamic diffusion (Fig. 4b). A significant interaction is observed between proteins and inhibitors (D2573, D2148, and D63). The quenching process was caused by the increased interaction, as indicated by the K_SV_ values (10^2^ to 10^5^ M^−1^) in Table 9. When inhibitors bind to proteins, the microenvironment around the binding site becomes less hydrophobic, exposing additional residues for interaction, which can be seen by greater values of binding sites (n).

Considering the cytotoxic effects of the lead molecules D2573, D2148, and D63 on human cells, NIH 3T3 cells were exposed to different concentrations (12.5 μg/mL, 25 μg/mL, and 50 μg/mL) of each sample. The cellular viability of samples was determined to be >75% after 24 h of incubation, even at a higher concentration (50 μg/mL), as indicated in the MTT assay (Fig. 6), showing that the lead molecules D2573, D2148, and D63 are nontoxic. Moreover, the *in vitro* results of IC_50_, enzyme kinetics, fluorescence, and the MTT assay are in harmony with *in silico* results obtained by virtual screening, molecular docking, and *in silico* pharmacokinetics.

**Conclusion.** This study identified three potential novel non-β-lactam broad-spectrum inhibitors (D2573, D2148, and D63), which specifically interact with the active site residues of selected β-lactamases. These active site residues are crucial for the binding and hydrolysis of β-lactam antibiotics. The identified β-lactamase inhibitors are competent to inhibit MBLs and SBLs simultaneously. Hence, these can be proposed as future molecules to form a single formulation of these inhibitors with an antibiotic to control the global multidrug resistance threat.

## MATERIALS AND METHODS

### Preparation of enzyme initial structure.

The 3D structures of SHV-1, TEM-1, CTX-M-15, KPC-2, NDM-1, and Amp-C, were retrieved from the Protein Data Bank (PDB no. 1SHV, 1XPB, 4HBU, 3DW0, 5ZGE and 4WYY), respectively (17–22). Discovery Studio 2.5 software was used to remove all water molecules and to add hydrogen atoms to the enzyme (23). To eliminate probable steric clashes and add hydrogen atoms, a minimization approach was applied. PyMOL software was used to create a surface model of all β-lactamases (24).

### Screening of the chemical database.

The PubChem database (25) was used to obtain 3D structures of one antibiotic (cefotaxime) and one β-lactamase inhibitor (avibactam). To find the hit compounds, the Maybridge database was utilized for screening compounds based on the attributes (Lipinski’s rule of five: CLogP, molecular weight, rotatable bonds, hydrogen bond donor, and hydrogen bond acceptor) of known inhibitors. Finally, about 11,000 compounds (out of a total of 16,000) were chosen. All the molecules had hydrogen atoms added to them. The DS 2.5 package was used to complete all the preparations. The minimization procedure was carried out with the Chem3D17.1 software MM2 energy minimization tool.

### Docking studies.

The chemical database was virtually screened using GOLD (Genetic Optimization for Ligand Docking) 5.0 (26). The van der Walls and hydrogen bonding and docking annealing parameters were set to 5.0 and 2.5, respectively. The parameters employed in the genetic algorithm were population size, 100; selection pressure, 1.2; number of operations, 1,00,000; number of islands, 5; niche size, 2; migrate; 10; mutate, 100; and crossover, 100. The GOLD fitness score, favorable binding, and molecular interactions with the active site amino acids were used to evaluate the docked compounds. The results were also validated using AutoDock Vina software (27). The compounds were screened using the GOLD fitness score and binding energy from AutoDock Vina.

### MD simulations.

GROMACS (Groningen Machine for Chemical Simulation) suite 5.0 (28, 29) and the GROMOS96 43a1 force field (30, 31) were used to run MD simulations for the protein-ligand complex. The PRODRG webserver was used to construct the ligand topologies (32, 33). Each docked complex was solvated with the extended simple point charge (SPC/E) water model in the cubic box, with a distance of 1.0 nm between the protein and the edge of the simulation box. Each system was balanced by introducing counterions and then reducing the amount of energy used. All of the systems were normalized after the topology generation by introducing Na+ or Cl^–^ ions as needed. Before proceeding with the MD simulations, the energy of each system was minimized.

The equilibration steps, that is, NVT (isothermal-isochoric) and then NPT (isothermal-isobaric) equilibration, were performed for 100 ps at constant volume, pressure (1 atm), and temperature (300 K) (34). After preparation, all the systems were ready to release the position restraints, and MD was run for 10 ns (Fig. 2).

D2573 showed the best binding energy and stability with the proteins of all the classes of β-lactamases as evident from molecular docking and 10-ns MD simulation. Finally, 50-ns production runs were carried out for D2573 and avibactam. Root mean square fluctuation (RMSF) and root mean square deviation (RMSD) were calculated using the gmx rmsf and gmx rms modules of GROMACS.

### *In silico* pharmacokinetic study of selected molecules.

Due to poor pharmacokinetics, the majority of medications identified during the drug discovery process were unable to pass human clinical trials. The absorption, distribution, metabolism, excretion, and toxicity (ADMET) parameters of pharmacokinetic research are critical descriptors for the human therapeutic use of any medication. These characteristics were calculated using ADMET modules in the Discovery Studio 3.5 program (Accelrys, USA). The SwissADME online service tool (http://swissadme.ch/) and PreADMET (http://preadmet.bmdrc.org/) were used to forecast the pharmacokinetics of selected ligands. Considering that 90% of orally active existing drugs/compounds fulfil Lipinski’s rule, the examined compounds were also assessed under Lipinski’s rule of five for oral bioavailability.

### Bacterial strain, antibiotics, and other reagents.

For cloning, E. coli DH5 and E. coli BL21(DE3) were employed. D63, D2148, D2573, and avibactam were purchased from Molport. Sigma (St. Louis, MO, USA) supplied the cefotaxime, imipenem, meropenem, and cefoxitin, and other chemicals and reagents were of analytical grade.

### Cloning of class A, B, and C type β-lactamases.

The plasmid DNA harboring *bla*_SHV-1_, *bla*_TEM-1_, *bla*_KPC-2_, and *bla_Amp-C_* genes were amplified by PCR using the following primers: SHV-1 forward (F) (5′ATATCATATGATGCGTTATATTCGCCTGTGT-3′), comprising the NdeI site, and SHV-1 reverse (R) (5′ATATAAGCTT TTAGCGTTGCCAGTGCTCGAT-3′), with the HindIII site, TEM-1 F (5′ATATCATATGATGAGTATTCAACATTTCCGTGTC-3′), comprising the NdeI site, and TEM-1 R (5′ATATAAGCTTGCTGCAATGATACCGCGAGACCCA-3′) with the HindIII site, KPC-2 F (5′ATATCATATGATGTCACTGTATCGCCGTCTA-3′), comprising the NdeI site, and KPC-2 R (5′ATATGGATCCTTACTGCCCGTTGACGCCCAATCC-3′) with the BamHI site, and Amp-C F (5′-ATCATATGTTCAAAACGACGCTCTG-3′), with the NdeI site, and Amp-C R (5′-ATGGTACCTTACTGTAGAGCGTTAAGAATC-3′), with the KpnI site. The *bla*_CTX-M-15_ and *bla*_NDM-1_ genes were cloned as previously described (35, 36). The PCR product was amplified using the above-listed primers. The GeneJET gel extraction kit was used to purify the PCR product according to the instruction handbook. With certain modifications, the digestion and ligation processes were carried out according to the enzyme manufacturer’s protocol (Fermentas, USA). T4 ligase enzyme was added to the digested product. Transformation of competent E. coli DH5 with the ligated product was done using the heat shock method. Transformants carrying the gene were chosen on LB agar plates containing ampicillin (100 μg/mL).

### Class A, B, and C type β-lactamase recombinant protein overexpression and purification.

Overexpression of proteins was done by adding 0.5 mM IPTG (isopropyl-β-d-thiogalactopyranoside), and purification of the NDM-1, SHV-1, TEM-1, CTX-M-15, KPC-2, and Amp-C recombinant protein was performed with an Ni-NTA affinity column. Purified protein was estimated to be more than 95% as determined by a single band of 28 to 34 kDa on SDS-PAGE according to the gene (NDM-1, SHV-1, CTX-M-15, TEM-1, KPC-2, and Amp-C). The final concentration of the protein was estimated with a NanoDrop instrument.

### Determination of MIC and minimum bactericidal concentration (MBC).

The MICs of various antibiotics in combination with selected inhibitors were determined on DH5α cells and clinical strains (AK-10, AK-12,AK-18, NP-6, AK-67, AK-66) using the microdilution method; Clinical and Laboratory Standards Institute (CLSI) guidelines were used to interpret the results (37, 38). In a series of 2-fold dilutions, E. coli cells were given escalating doses of antibiotics from 0 to 512 μg/mL. The MIC was calculated as the lowest concentration at which observable bacterial growth is completely inhibited.

Following the measurement of the MIC, aliquots of 50 μL from all wells with no obvious bacterial growth were seeded on brain heart infusion (BHI) agar plates and incubated for 24 h at 37°C. The MBC endpoint is reached when 99.9% of the bacterial population is killed by the lowest concentration of an antimicrobial agent. This was accomplished by monitoring the presence or absence of bacteria on pre- and postincubated agar plates.

### Determination of inhibition constant (*K_i_*) and IC_50_ values.

The direct competition between the β-lactamase substrate, nitrocefin, and their inhibitors were used to determine the IC_50_ and *K_i_* values. Using a Shimadzu UV-VIS spectrophotometer UV-1800, variable quantities of inhibitor D63, D2148, and D2573 (0 to 3 μM), fixed concentrations of purified proteins (1 nM), and nitrocefin substrate (100 μM) were used in a reaction at 486 nm. Plotting the percent residual enzyme activity on nitrocefin versus the inhibitor concentration (log_10_) yielded the IC_50_ values. The IC_50_ value is the inhibitor concentration that inhibits enzyme hydrolytic activity by 50% (50% inhibitory concentration).

The inhibition constant, *K_i_*, was calculated from the IC_50_ value by applying the Cheng-Prusoff correction (equation 1; 39).

(1) $$K_{i}\;={\text{ IC}}_{50}/\left(\text{1}+\text{ S}/K_{m}\text{NCF}\right)$$

where *K_m_* and S are the Michaelis-Menten constant and concentration of nitrocefin, respectively.

### Steady-state and kinetics parameter.

The steady-state kinetics of all six proteins were determined using the antibiotics:imipenem (Δε295 = −11,500 M^−1 ^cm^−1^), meropenem (Δε297 = −10,940 M^−1 ^cm^−1^), and cefotaxime (Δε264 = −27,250 M^−1 ^cm^−1^). In this study, the D63, D2148, and D2573 inhibitors were tested. Hydrolysis of β-lactam antibiotics was detected by monitoring the variation in absorbance due to cleavage of the β-lactam ring in 50 mM, pH 7.0, phosphate buffer (40, 41). For hydrolysis of substrate (cefotaxime, imipenem, and meropenem) kinetics parameters, *K_m_* and *k*_cat_ were calculated by using Michaelis Menten equations (equations 2 and 3).

(2) $$V\;=V_{\max}\;\left[\text{S}\right]/K_{m}+\;\left[\text{S}\right]$$

(3) $$k_{\text{cat}}\;=\;V_{\max}/\text{E}$$

where E and S are the enzyme and substrate concentration, *K_m_* is the Michaelis-Mentent constant, *k*_cat_ is the turnover number, and *V* and *V*_max_ are the initial and maximum velocity of the hydrolysis, respectively.

### Fluorescence spectra measurements.

A Shimadzu RF-5301PC spectrofluorometer (Shimadzu Corporation, Kyoto, Japan) was used to measure the fluorescence spectra. Quenching was observed by measuring intrinsic fluorescence at 295 nm between 300 and 400 nm. The emission and excitation slits were both adjusted to 5 nm. Then, 2 μM each D2573, D2148, and D63 were added to a 3-mL sample containing 2 μM protein (final volume, 30 μL). All the fluorescence intensities were corrected for the inner filter effect. The determination of the number of binding sites (*n*) and binding constant (Kb) at 298 K temperature for all the protein-inhibitor combinations from the Stern-Volmer equation and modified Stern-Volmer equation (equations 4 and 5).

(4) (F0)/F = 1 +  Ksv[Q]

(5) $$\text{Log }\left({\text{F}}_{0}-\text{ F}\right)/\text{F}=\text{ logKb }+\text{ n log}\left[\text{Q}\right]$$

Fo and F are the fluorescence intensities in the absence and presence of quencher (D63, D2148, D2573).

### MTT assay.

A cytotoxicity experiment was performed on NIH 3T3 cells. The NIH 3T3 cells were grown at 37°C in Dulbecco’s modified Eagle’s medium (DMEM) supplemented with 10% fetal bovine serum (FBS) and 1% antibiotic, penicillin-streptomycin (pen-strep), in a humidified incubator with 5% CO_2_. Sterile conditions were maintained at all times. Cells (~10^5^ cells/well) were seeded in 96-well plates and allowed to adhere overnight. The cells were subsequently given different quantities of the lead compounds D2573, D2148, and D63 (12.5 μg/mL, 25 μg/mL, and 50 μg/mL, respectively). A conventional 3-(4,5-dimethylthiazol-2-yl)-2,5-diphenyl tetrazolium bromide (MTT) test was used to measure cell survival after 24 h. The supernatants were removed, and each well was filled with 90 μL of new medium and 10 μL of MTT (1 mg/mL) solution, which was then incubated for 4 h at 37°C. The formazan crystals generated during the reduction of MTT were dissolved in 150 μL of dimethyl sulfoxide (DMSO), and the absorbance was measured by measuring the optical density using a spectrophotometer at a wavelength of 570 nm.
